# Dynamic model of respiratory infectious disease transmission in urban public transportation systems

**DOI:** 10.1016/j.heliyon.2023.e14500

**Published:** 2023-03-11

**Authors:** Zuiyuan Guo, Guangquan Xiao, Yayu Wang, Sidong Li, Jianhong Du, Botao Dai, Lili Gong, Dan Xiao

**Affiliations:** a Department of Infectious Disease Prevention and Control, PLA Northern Theater Command Center for Disease Control and Prevention, Shenyang, China; b Training Base of Non-Commissioned Officer Specialized in Aviation Support of Naval Aeronautical University, Qingdao, China; c Liaoning Agricultural Development Service Center, Shenyang, China; d Department of Psychiatry, General Hospital of Northern Theater Command, Shenyang, China; e China National Clinical Research Center for Neurological Diseases, Beijing Tiantan Hospital, Beijing, China

**Keywords:** Dynamic model, Public transportation system, Respiratory infectious disease

## Abstract

During the epidemics of respiratory infectious diseases, the use of public transportation increases the risk of disease transmission. Therefore, we established a dynamic model to provide an in-depth understanding of the mechanism of epidemic spread via this route. We designed a computer program to model a rail transit system including four transit lines in a small town in which assumed 70% of the residents commute via these trams in weekdays and the remaining residents take the tram at random. The model could identify the best travel route for each passenger and the specific passengers onboard when the tram passed through each station, and simulate the dynamic spread of a respiratory pathogen as the passengers used the rail transit system. Based on the program operating, we estimated that all residents in the town were ultimately infected, including 86.6% who were infected due to the public transportation system. The remaining individuals were infected at home. As the infection rate increased, the number of infected individuals increased more rapidly. Reducing the frequency of trams, driving private cars or riding bicycles, showing nucleic acid certificates and wearing masks for passengers, etc., are effective measures for the prevention of the spread of epidemic diseases.

## 1 Introduction

Respiratory infectious diseases can spread rapidly in crowded places, leading to outbreaks and epidemics, such as H1N1 and COVID-19 [[1], [2], [3]], etc. The convenience of public transportation makes it preferred mode of travel in many Chinese metropolises with developed economies and congested roads [4]. However, while public transportation systems provide convenient travel, they are a means of spread for some respiratory infectious diseases and may contribute to epidemics as susceptibles (S), exposers without infectiousness in the incubation period (E), infectors with infectiousness (I), and those recovered individuals with immunity (R), which may interact when using public transportation [[5], [6], [7]]. Pathogens are transmitted from I to S due to close contact between passengers, and epidemics occur due to the continuous flow of passengers. In addition, residents who become infectious in the community can transmit pathogens to their families when at home [[8], [9], [10]]. If these infectious family members take public transportation, pathogens can continue to spread.

In order to deeply understand the mechanism of respiratory infectious diseases transmitted by urban public transport system, it is necessary to establish a dynamic model. Classical compartment models established using ordinary differential equation sets are the most commonly used to quantitatively analyze and predict the epidemic trend of infectious diseases. Several previous studies have used these models to analyze the epidemic characteristics of respiratory infectious diseases [[11], [12], [13], [14], [15]]. However, passengers' dynamic taking public transportation systems cannot be analyzed using differential equations, because each passenger's route and travel time as well as the specific passengers simultaneously in a carriage are random. Therefore, the classical dynamic model is not applicable here. In order to solve this problem, it is necessary to establish a random individual-based dynamic model that can accurately describe the specific route of each passenger, which can simulate the dynamic process of an infectious disease epidemic caused by the continuous flow and mutual contact of people in the carriage.

Many researchers have established individual-based models that have been used by governments and international health organizations to devise containment strategies for smallpox, MRSA, dengue, mouth disease, and influenza [[16], [17], [18], [19]]. In addition, some open-source platforms, such as global-scale agent model developed by Johns Hopkins University [19], the framework for reconstructing epidemic dynamics developed by University of Pittsburgh [20], and COVID-19 agent-based simulator (Covasim) developed jointly by several research institutions [21], have been utilized for analyzing pandemic influenza and COVID-19. Particularly, some of these models focused on analyzing the role of transportation in disease transmission. For example, Cooley established a computer simulation of New York City's five boroughs that incorporated subway ridership into an SEIR model [22]; Zhang established a large-scale individual-based model for epidemic prediction in the context of the metropolitan area of Beijing China, where a microscopic public transport system (including metros and trams) is simulated [7]; Li examined the risk of transmission of COVID-19 between subway commuters using the SEIR model [23], etc. These models have played a positive role in discussing the epidemic transmitted by public transport. However, these studies did not focus on revealing the epidemiological law of disease transmission on the basis of simulating the flow of each passenger in the transmit lines.

We have developed several individual-based dynamic computer models by designing computer program code (the stochastic collision model) that was created to study the transmission mechanism of infectious diseases among people. For example, it has been used to analyze the spread of adenovirus in the army [24], the spread of COVID-19 in China at the beginning of 2020 [25], epidemics in the community caused by asymptomatic SARS-CoV-2 infectors [26], and the spread of respiratory infectious diseases through family feasts in a complex network [27] and population mobility based on city networks [28]. These research practices adequately show that the random collision model can not only accurately describe the activity state of individuals from a micro perspective, but also show the transmission process of the epidemic in the population from a macro perspective. It has good flexibility and applicability, which is a powerful tool to study the transmission mechanism of infectious diseases in complex conditions. The stochastic collision model can integrate infectious disease dynamics with a wider range of disciplines, and further enrich and expand the research scope of the dynamics model.

Compared with our previous studies, the main contributions of this model are as follows: (1) the place where people contact is in the public tram carriage rather than the daily contact, which further expand the research scope of dynamic model; (2) based on the innovation of the algorithm, we can calculate the dynamic changes of passengers in the carriage when the tram passes through each station, and simulate the spread of infectious diseases according to the dynamic passengers; (3) this model is conducive to the public's deep understanding of the risks of respiratory infectious diseases spread by urban public transportation systems and provides a scientific basis for governments to formulate prevention and control measures.

## 2 Methods

### 2.1 Prerequisites used to establish the model

To make the model more concise and improve the efficiency of the computer program, several preconditions were adopted.

First, the model was based on an epidemic occurred in a virtual small, secluded town with 5680 population and 2000 families, the composition of household population was listed in Table 1. Migration, birth, and death were not considered in the model because the epidemic period is relatively short. All of the town's residents were susceptible to the pathogen at the start of the epidemic.

**Table 1:** Model parameters.

Description	Distribution characteristics	Numerical values	Sources
Incubation period 1/φ	Lognormal distribution	μ=5.2 σ=0.87	[32]
Infectious period 1/γ (mean duration from onset to hospital admission; estimated to be 12.5 days; 95% CI, 10.3–14.8 days)	Weibull distribution	Shape parameter m=1.66 Scale parameter η=8.73	[32]
Duration of hospitalization 1/φ	Uniform distribution	12–20 days	[33]
The probability that an S is infected after traveling from one tram stop to the next with an I in the carriage *β*	Constants	0.1, 0.2, 0.3	Assumed
Total number of families in the small town	Constant	2000	Assumed
Probability that residents are commuters *q*	Constant	0.7	Assumed
Probability of residents taking trams at random *p*	Constant	0.5	Assumed
Frequency of taking trams at random *λ*	Constant	2	Assumed
Distribution of Chinese family sizes	Constants	Probabilities that the family size was 1, 2, 3, 4, 5, or 6 were 0.18, 0.3, 0.22, 0.16, 0.08, and 0.06, respectively	[34]

Abbreviations: CI, confidence interval.

**Fig. 1:**
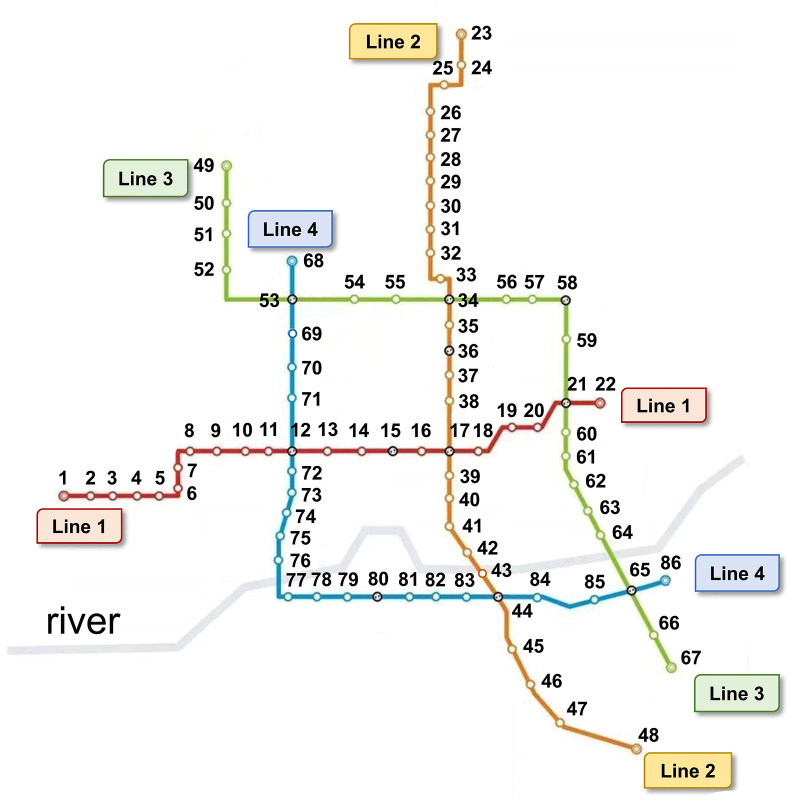
**Planar graph of the rail transit network.** The rail transit network includes four tram lines and 86 stations. The forward and backward directions of each line are designated.

Second, this model was based on the presence of four rail transit lines (Fig. 1). As rail transit is not affected by road traffic conditions, the trams can arrive at stations on time. The stations were identified by indexes, and a total of 86 stations and seven junctions were included in the rail transit system in this model. Each rail transit line was considered to be able to move in the forward (serial station indexes from small to large) and backward (serial station indexes from large to small) directions. The departure time of the first train was 6:00 a.m., and the last train departed at 9:00 p.m. each day, with a departure frequency of once every 5 min. The driving time between any two adjacent stations, including the stopping time at the previous station, was 3 min. The transfer time of passengers at transfer stations was 1 min.

Third, it was assumed that 70% of the town's residents were commuters who chose to go to and from work (or school) using this public transportation system. The working and school days were Monday through Friday, with peak travel occurring from 6:30–8:30 a.m. and 5:00–7:00 p.m. One time point was randomly chosen from each of the two peak time periods each day for each commuter. In this model, commuters only took public transportation when they went to and from work (or school) on weekdays without driving private cars. We assumed the remaining 30% of residents had a 50% probability of taking the trams at any time each weekday. During the weekend, commuters and non-commuters had a 50% probability of taking public transportation at any time. Regardless of the weekdays or the weekends, the number of passengers taking public transportation randomly shows a Poisson distribution with a mean of 2 per day, and the starting and ending stations were randomly selected, though the commuters' routes to work were fixed. Commuters in the same family had the same starting station when they went to work (or school).

### 2.2 Selection of optimal travel routes

**Fig. 2:**
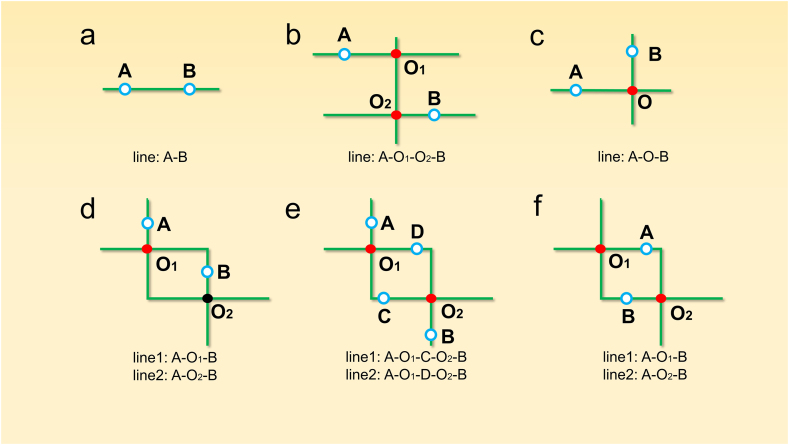
**Rail transit line transfer schemes.** a. The starting station (A) and the ending station (B) are on the same line. b. A and B are not on the same line, and there is no intersection point between the two lines. c. The lines of A and B intersect once. d, e and f. The lines of A and B intersect twice.

Instead of using the classical graph theory to calculate the optimal route, a more concise and efficient algorithm based on the characteristics of the rail transit networks shown in Fig. 2 was designed. In summary, it was first checked if the starting and ending stations were on the same transportation line. If so, the optimal route was determined immediately (Fig. 2a). When the starting and ending stations were not on the same transportation line, the route with the least number of stops and no more than two transfers was defined as the optimal route. When the starting station's line did not directly intersect with the ending station's line, two transfers were necessary (Fig. 2b). When the starting station's line intersected with the ending station's line, only one transfer was necessary (Fig. 2c). When the starting and ending stations' lines intersected twice (which occurred with Lines 3 and 4), only one transfer was necessary, but there were three possible routes (Fig. 2d, e, and 2f). In this study, only paths with two or fewer transfers were considered. Therefore, before determining the optimal route from the starting station to the ending station, all the routes that required one or two midway transfers were identified and the route with fewest stops was determined as the optimal route. The algorithm is detailed in the study appendix (lines 92–172).

### 2.3 Distribution of passengers on rail transit lines

Firstly, the departure and arrival times from any station of each tram on each line were set. Then, based on each passenger's optimal travel route and arrival time at the starting station, the times for the passengers to get on the tram at the starting station, get off at the transfer station, get on another tram at the transfer station, and get off at the ending station were calculated. Finally, after the above time information of all passengers is summarized, we can determine the indexes of passengers on each tram at any time on any day. The specific algorithm is shown in the appendix (lines 216–516).

### 2.4 Spread of pathogens on trams

Passengers were divided into S, E, I, R that were constantly changing and stored in a data frame. S became E after infected by I. After the incubation period, E became I. The infectious period was defined as the period from disease onset to hospital admission. Patients during hospitalization (H) did not use public transportation. After recovery, H became recovered and immunized and started using the public transportation again. As passengers got on and off at each station, the passengers on the trams changed constantly. Therefore, according to the previous calculation, we can get all the passengers indexes and their infection status in the carriage when each tram passed through a station. It was feasible to simulate the epidemic spreading in the carriage. The average infection rate was assumed to be *β* when an S came into contact with an I on the tram from one station to the next. When the number of I in the tram was *m*, the probability of an S becoming infected was *1-(1-β)*^*m*^. Therefore, the number of new E could be determined every time the tram passed through a station. Thereafter, the infection status of passengers should be updated. The specific algorithm is explained in the appendix (lines 517–550).

### 2.5 Spread of viruses within the family

Besides the trams, the pathogen was able to spread in the family when an I was at home. It was assumed that once an E of a family became an I, all susceptible family members would be infected that night. Because people are generally susceptible to this pathogen, and they contact closely at home for a long time at night. The specific algorithm is explained in the appendix (lines 551–584).

**Fig. 3:**
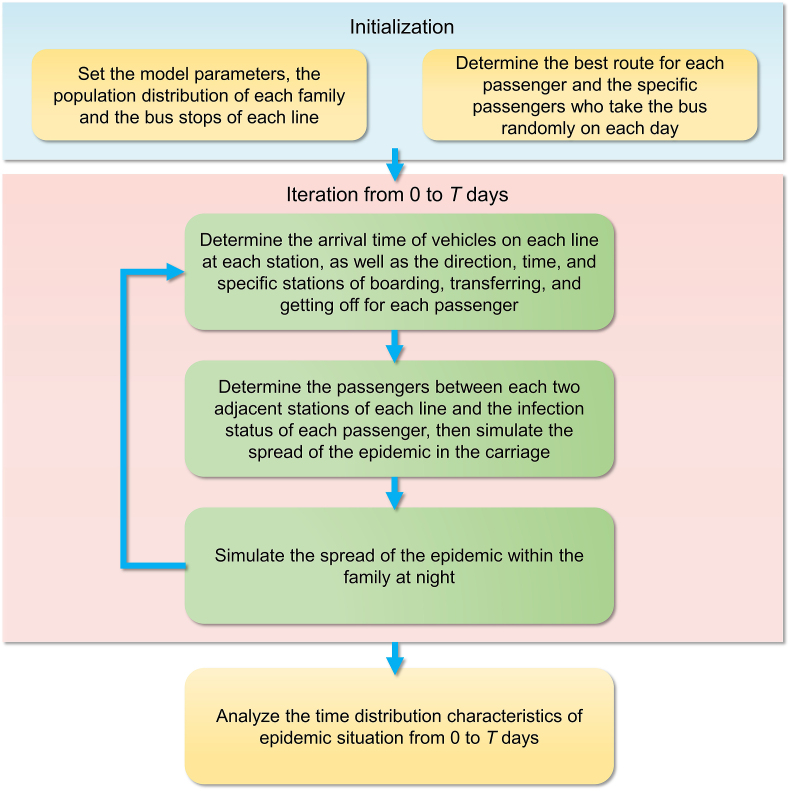
Model design framework.

In this study, COVID-19 was used as an example of infectious respiratory disease. The model parameters are listed in Table 1. Median and interquartile values (25%–75%) were calculated based on 50 trials of the program. The design framework of the model is demonstrated in Fig. 3.

Because the individual behavior and disease course is random, the operation results of S, E, I, and R in each iteration are uncertain. However, the quantity of these populations can be reflected by the following equation (1) which are not calculation tools though.

(1) $$\begin{array}{l} △S=-\sum_{i}\sum_{j}\sum_{k}X_{ijk}^{s}-\sum_{m}\delta S_{m}^{h}\\ △E=\sum_{i}\sum_{j}\sum_{k}X_{ijk}^{s}+\sum_{m}\delta S_{m}^{h}-\varphi E\\ △I=\varphi E-\gamma I\\ △H=\gamma I-\varphi H\\ △R=\varphi H\\ where,X_{ijk}^{s}∼B{\left(S_{ijk}^{s},1-(1-\beta \right)}^{I_{ijk}^{s}}) \end{array}$$

△ indicates the variations of population on day *d*. $X^{s}$ indicates the number of *S*→*E* in the carriage during the tram is running between two adjacent stations, and it follows the binomial distribution with $n=S^{s}$, $p=1-{\left(1-\beta \right)}^{I^{s}}$; *k, j, i* indicate the order number of stations, trams, and lines, respectively. $\sum_{i}\sum_{j}\sum_{k}X_{ijk}^{s}$ indicates the total number of *S*→*E* in the trams.

δ indicates whether there emerged an *E*→*I* in the *m*-th family for the first time on *d*-th day (1: yes, 0: no); $S_{m}^{h}$ indicates the number of *S* in the *m*-th family, and $\sum_{m}\delta S_{m}^{h}$ indicates the total number of *S*→*E* in the family. Parameters φ, γ, φ are explained in Table 1.

### 2.6 Sensitivity analyses

Since actual epidemic data was composed of diverse modes of transmission besides the public transport system, insufficient data could be used to calibrate and fit with the model. Sensitivity analyses were performed to test the reliability and rationality of the model in terms of four important parameters of the model—the probability that residents are commuters (*q)*, the probability of infection (*β)*, the probability of residents taking trams at random (*p*), and the frequency of taking trams at random (*λ)*.

We applied Partial rank correlation coefficients and Latin hypercube sampling (PRCC-LHS) that is a developed and extensively used algorithm to sensitivity analysis. The correlations between a set of parameters and the model outputs after removing the linear effects of the target parameter were calculated [29]. Continuous time PRCC-LHS was used for four main parameters in this study. Each parameter interval was divided into *N* smaller and equal intervals. Then randomly selected one sample from each interval. Further, these selected parameter samples were included in the model to work out the outputs at each time point [29,30]. A series of standard coefficients denoting the correlation between each parameter and the model output were calculated. All analyses were conducted using MATLAB R2019a software (MathWorks, Natick, Massachusetts, USA).

## 3 Results

### 3.1 Passenger volumes

**Fig. 4:**
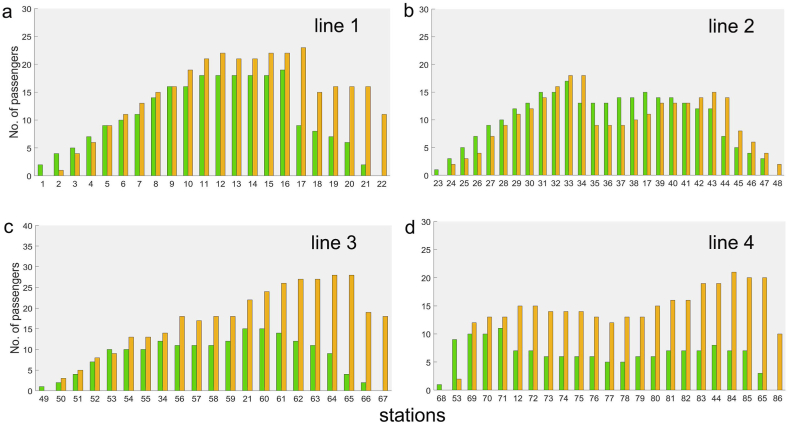
**Number of passengers on each transit line after one tram passing through each station.** The number of passengers traveling in the forward direction (increasing station numbers) is shown in the yellow histogram, while the green histogram shows the number of passengers traveling in the backward direction (decreasing station numbers). Graphs a, b, c, and d represent Lines 1, 2, 3, and 4, respectively.

When the trams departed at 7:00 a.m. on the first working day, the number of passengers in the carriage on each line at each station was calculated (Fig. 4a, b, 4c, and 4d). Due to the asymmetric topology of the rail transit line system, the numbers of passengers traveling forward and backward were different on Lines 3 and 4, comparatively, they were similar on Lines 1 and 2.

### 3.2 Infected passengers on each rail transit line

**Fig. 5:**
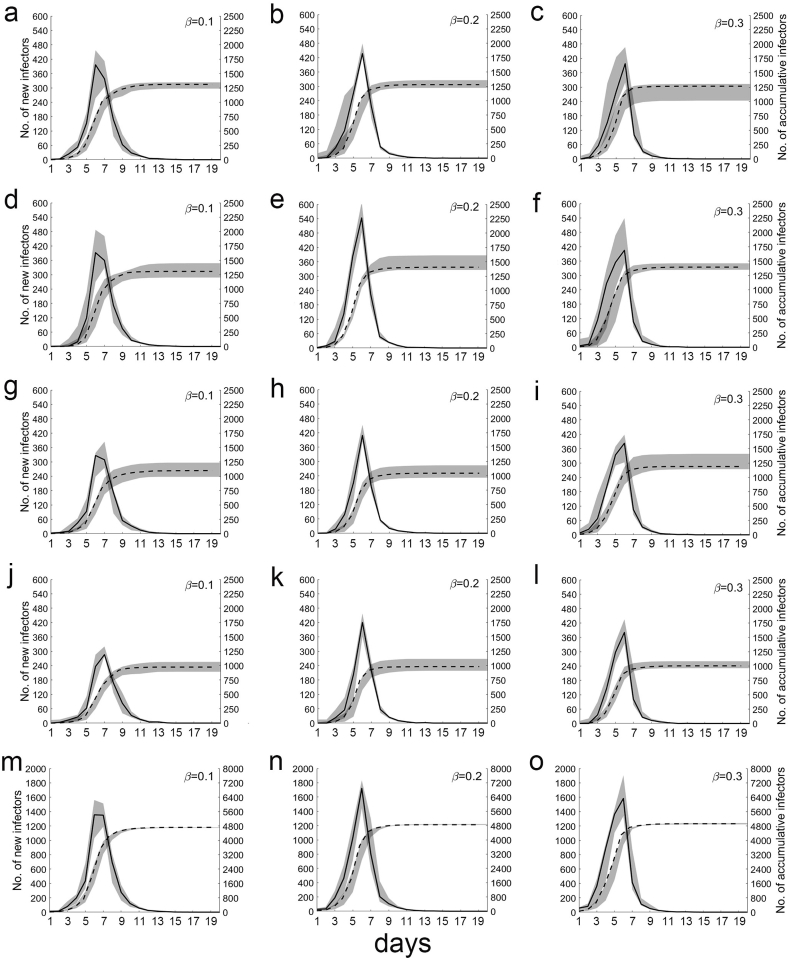
**Number of new and cumulative infected individuals spread via public transportation.** The median number of new infected individuals, which is measured using the left coordinate axis, is shown with a solid line. The median cumulative number of infected individuals, which is measured using the right coordinate axis, is shown with a dotted line. The shaded area represents the interquartile range (25%–75%) of infected individuals. Letters a, b, and c represent Line 1; d, e, and f represent Line 2; g, h, and i represent Line 3; j, k, and l represent Line 4; and m, n, and o represent the sum of the four lines.

We assumed that there was one source of infection with *t* = 0. On Line 1, the median peak number of new infected individuals (E) were 389, 437, and 398, the median cumulative number of E on the twentieth day were 1,273, 1,280, and 1266 when *β* was 0.1, 0.2, and 0.3, respectively (Fig. 5a, b, and 5c).

On Line 2, the median peak number of new E were 392, 544, and 405, the median cumulative number of E on the twentieth day were 1,307, 1,407, and 1395 when *β* was 0.1, 0.2, and 0.3, respectively (Fig. 5d, e, and 5f).

On Line 3, the median peak number of new E were 327, 409, and 383, the median cumulative number of E on the twentieth day were 1,098, 1,046, and 1188 when *β* was 0.1, 0.2, and 0.3, respectively (Fig. 5g, h, and 5i).

On Line 4, the median peak number of new E were 286, 421, and 381 the median cumulative number of E on the twentieth day were 975, 984, and 1004 when *β* was 0.1, 0.2, and 0.3, respectively (Fig. 5j, k, and 5l).

Overall, the median peak number of new E on all rail transit lines were 1,356, 1,855, and 1,584, the median cumulative number of E on the twentieth day were 4,722, 4,889, and 4,928, when *β* was 0.1, 0.2, and 0.3, respectively (Fig. 5m, n, and 5o). The above results are listed in Table 2.

**Table 2:** Distribution of new and accumulated infected individuals in each line.

	Line 1	Line 2	Line 3	Line 4	Total
*β*=0.1	389 (261–453) 975 (894–1065)	392 (328–487) 1307 (1200–1449)	327 (275–384) 1098 (989–1235)	286 (281–320) 975 (895–1065)	1356 (1190–1561) 4722 (4695–4725)
*β*=0.2	437 (426–479) 1280 (1224–1358)	544 (483–610) 1407 (1359–1612)	327 (275–384) 1046 (968–1183)	421 (361–458) 984 (911–1118)	1855 (1743–1910) 4889 (4878–4910)
*β*=0.3	398 (319–467) 1266 (1015–1305)	405 (351–539) 1395 (1349–1461)	383 (334–435) 1188 (1142–1409)	381 (334–435) 1004 (962–1084)	1584 (1338–1904) 4928 (4891–4960)

The first row of the cell represents the median when the new infected individuals reached the peak with the fluctuation range of 25–75%. The second row shows the median cumulative infected individuals and the fluctuation range of 25–75% on the 20th day.

### 3.3 Temporal distribution of existing individuals based on infection status

**Fig. 6:**
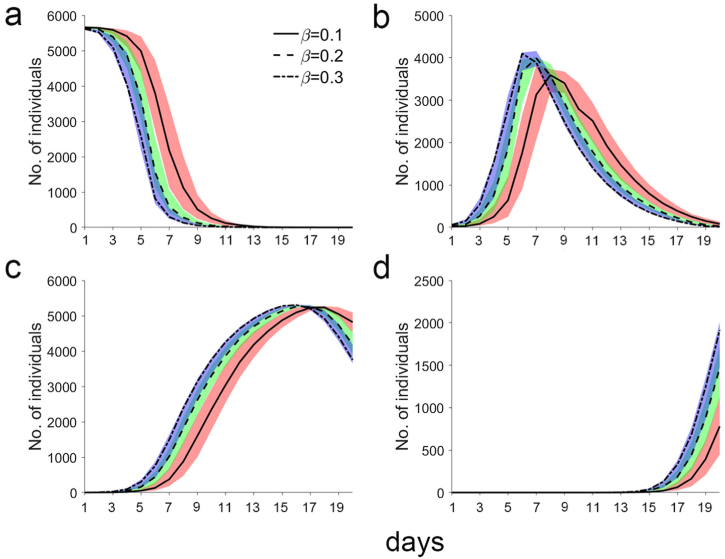
**Temporal distribution of the existing population's infection status. a, b, c, d represent the existing number of S, E, I, R, respectively.** The median number of people is shown by the black solid and dotted lines. The red, green, and blue areas represent the interquartile ranges (25%–75%) of the population when *β* = 0.1, 0.2, and 0.3, respectively.

The existing number of S decreased rapidly over time (Fig. 6a). When *β* was 0.1, 0.2, and 0.3, the simulation reached zero on the eighteenth, fifteenth, and thirteenth days, respectively. This means that all residents of the town were infected. The number of E initially increased and then decreased (Fig. 6b). When *β* was 0.1, 0.2, and 0.3, the number peaked on the eighth, seventh, and sixth days, respectively, and the median peak number were 3,592, 3,990, and 4,100, respectively. The number of I also increased before decreasing (Fig. 6c). When *β* was 0.1, 0.2, and 0.3, the number peaked on the eighteenth, seventeenth, and sixteenth days, respectively, and the median peak number were 5,236, 5,248, and 5,307, respectively. The number of R increased rapidly in the later epidemic stage (Fig. 6d). When *β* was 0.1, 0.2, and 0.3, the number began to increase on the thirteenth, thirteenth, and twelfth days, respectively, and the median number reached 780, 1,470, and 1,915, respectively, on the twentieth day.

### 3.4 Sensitivity analyses

**Fig. 7:**
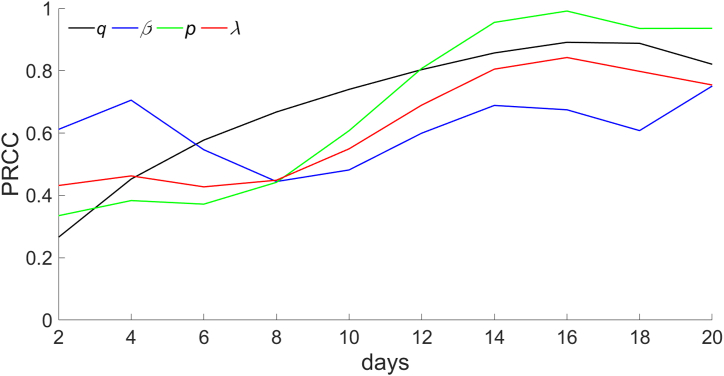
**Sensitivity analysis of continuous time.***q,* the probability that residents are commuters; *β,* the infection rate that an S is infected by an I in the carriage during the tram is traveling from one station to the next; *p,* the probability of residents taking trams at random; and *λ,* the frequency of taking trams at random.

In this study, sensitivity analyses were conducted with the model based on four parameters (*q*, *β*, *p*, *λ*) and a continuous-time series for the total four lines' cumulative number of infected individuals. We considered 50 samples from a uniform distribution for each parameter range. PRCCs near 1 indicate that the parameter has a more positive affect on the output. In contrast, a value closer to 0 indicates that the output result is less affected by the parameter (Fig. 7). The results reflected that these four parameters are positive correlated with the cumulative number of infected individuals during 0–20 days, and the correlation coefficients increase gradually with time. Since the larger the *q* is, the more is the number of commuters taking the tram, and the more is the number of infected individuals; the greater the *β* is, the larger is the probability of being infected when riding, and the higher is the number of infected individuals. The greater is the probability *p* and frequency *λ* of random ride, the more is the number of people who will ride randomly, and the more is the number of infected individuals. Sensitivity analysis reasonably explains the relationship between important parameters and output results and confirms the scientific validity and stability of the model.

## 4 Discussion

### 4.1 Innovations

The innovation of this study is mainly reflected in three aspects. Firstly, the model that combines traffic engineering with infectious disease dynamics was used to study disease transmission in public transportation systems. This model overcomes the limitation of traditional dynamic models that determine disease transmission through daily contact. Secondly, the process of disease transmission is completely controlled by a computer program, allowing for richer and finer pre-determined details, which enhances the flexibility of the model. For example, the starting and ending stations of passengers and the operation times of the rail transit system are pre-determined. Thirdly, the processes of disease spread during the day (within the public transportation system) and at night (within the homes) are discussed separately, which is consistent with real-life disease transmission.

### 4.2 Selection of optimal routes

The classic Dijkstra algorithm was not used in this study to select optimal routes for passengers due to the particularity of node distribution and connectivity in the rail transit system [31]. If the classic Dijkstra algorithm is used for analysis, a complex data structure model of the rail transit system diagram would be necessary. In Fig. 1, any two stations can be connected by one or two transfers. Therefore, an algorithm to identify the transfer station was designed. In this algorithm, the combinations of no more than two transfer stations connecting the starting station and the ending station were identified, then all routes passing through transfer stations were listed, and the route with the least total number of stations was defined as the optimal route. This algorithm is simpler to program and requires fewer computations.

### 4.3 Prevalence trends of epidemics

Regardless of the value of *β*, the existing number of E increased rapidly before decreasing rapidly in this model (Fig. 6b). The rapid increase is due to the sharp increase in the number of I (the sources of infection). Theoretically, when the product of the number of S and I reaches a maximum, the growth rate of E is at the maximum. Thereafter, the existing number of E decreases rapidly due to the rapid decrease in the number of S (Fig. 6a). On the twentieth day, the cumulative number of E was approximately 86.6% of the total population (which is 6.5 times the rate via family transmission) and was not significantly different irrespective of the values of *β* (Fig. 5m, n, and 5o). These results suggest that transmission via public transportation systems is the main mechanism for the spread of epidemics.

### 4.4 Limitations

This study has some limitations. Firstly, real-life data were not available for model fitting, which affects the scientific validity of the model to a certain extent. Because we do not have access to the relevant data and literature on the transmission of respiratory infectious diseases through public transportation systems. Additionally, an epidemiological investigation regarding this type of spread would be challenging. Therefore, the model was designed based on the general rules of urban rail transit system operation and passenger travel. Secondly, this model only focuses on public transportation systems and homes as the places where epidemics originate and does not consider other modes of transmission, such as daily contacts. Although this assumption facilitates the quantitative analysis, it does not reflect real-world conditions. Thirdly, the impact of other travel modes on the spread of epidemics was not considered despite the fact that passengers travel via several modes of public transportation in the real world, which will make the prediction results deviate from reality. Fourthly, this study did not discuss the control and prevention measures in the model, such as restricting residents using the public transport system, wearing masks during riding, and people working at home during the epidemic. Even with these limitations, this study reflects the role of the public transportation system in the spread of epidemics and provides a new exploration of dynamic models in the field of public health.

## 5 Conclusion

In summary, we simulated a dynamic process of passengers’ flow in the tram system to spread respiratory infectious diseases by designing an individual-based computer model and quantitatively analyzed the time distribution of the number of passengers in different infection states. Compared with previous models, this study focuses on the role of urban public transport system in the process of disease transmission. The random collision model used in this study can accurately describe the detailed process; comprehensively show the process of occurrence, development, and disappearance of infectious diseases; reveal the mechanism of infectious diseases transmitted by public transport system; and further enrich and develop the infectious disease transmission dynamics. The study found that respiratory infectious diseases can spread rapidly through urban public transport system. Therefore, when the epidemic breaks out, the government should appropriately reduce the number of trams, remind passengers to wear masks, and encourage residents to drive private cars, take taxies or ride bicycles. These measures can effectively slow down the spread of the epidemic.

## Ethics approval and consent to participate

This is a computational model study and does not involve human trials. Thus, an institutional review board statement is not provided.

## Consent for publication

Not applicable.

## Availability of data and materials

All program code generated or analyzed during this study are included in this published article [and its supplementary information files].

## Author contribution statement

Zuiyuan Guo: Conceived and designed the experiments; Performed the experiments; Analyzed and interpreted the data; Contributed reagents, materials, analysis tools or data; Wrote the paper.

Guangquan Xiao; Yayu Wang; Jianhong Du; Botao Dai; Lili Gong; Dan Xiao; Sidong Li: Contributed reagents, materials, analysis tools or data.

## Funding statement

This research did not receive any specific grant from funding agencies in the public, commercial, or not-for-profit sectors.

## Data availability statement

Data associated with this study has been deposited at the Zenodo (doi: 10.5281/zenodo.7008609).

## Declaration of interest’s statement

The authors declare no competing interests.
